# Perioperative complications in native nephrectomy as a therapeutic approach for symptomatic polycystic kidney disease: a comprehensive study

**DOI:** 10.1007/s11255-026-05155-8

**Published:** 2026-04-21

**Authors:** Alireza Helal Birjandi, Nicolas Richter, Pouriya Faraj Tabrizi, Hamza Idais, Markus A. Kuczyk, Hossein Tezval

**Affiliations:** 1 Department of Urology and Urological Oncology, Hannover Medical School (MHH), Hanover, Germany; 2 Department of General, Visceral and Transplant Surgery, Hannover Medical School (MHH), Hanover, Germany

**Keywords:** ADPKD, Native nephrectomy, Perioperative complications, Kidney transplantation, Minimally invasive surgery

## Abstract

**Purpose:**

To assess perioperative complications after native nephrectomy in autosomal dominant polycystic kidney disease (ADPKD), identify risk factors and evaluate surgical timing and approach.

**Methods:**

We retrospectively analyzed 124 nephrectomy episodes in patients with ADPKD treated at a single tertiary center between October 2012 and October 2022. Complications were graded according to the Clavien–Dindo classification. Multivariable logistic regression was used to assess predictors of overall and major complications.

**Results:**

Perioperative complications occurred in 73/124 episodes (58.9%), including 31 major complications (25.0%). Lower preoperative hemoglobin independently predicted overall complications (OR 0.74 per g/dL, 95% CI 0.61–0.89; *p* = 0.002), whereas left-sided nephrectomy was associated with a lower overall complication risk (OR 0.25, 95% CI 0.09–0.72; *p* = 0.011). Diverticulosis independently predicted major complications (OR 5.03, 95% CI 1.76–14.33; *p* = 0.003). Simultaneous bilateral nephrectomy showed the highest overall complication rate (69.7%). Open procedures were performed for markedly larger kidneys than minimally invasive procedures (median operative specimen weight 3518.5 g vs 495.5 g; *p* < 0.001). Minimally invasive surgery was not independently associated with a lower complication risk (OR 0.75, 95% CI 0.15–3.79; *p* = 0.726).

**Conclusion:**

Native nephrectomy in ADPKD is associated with substantial perioperative morbidity and should remain restricted to carefully selected patients. Surgical timing relative to transplantation should be guided primarily by symptoms and clinical necessity. Simultaneous bilateral nephrectomy appears to carry the greatest perioperative burden. Although minimally invasive surgery is feasible in selected patients with smaller kidneys, our data do not demonstrate an independent reduction in morbidity after adjustment for case complexity.

## Introduction

**Fig. 1:**
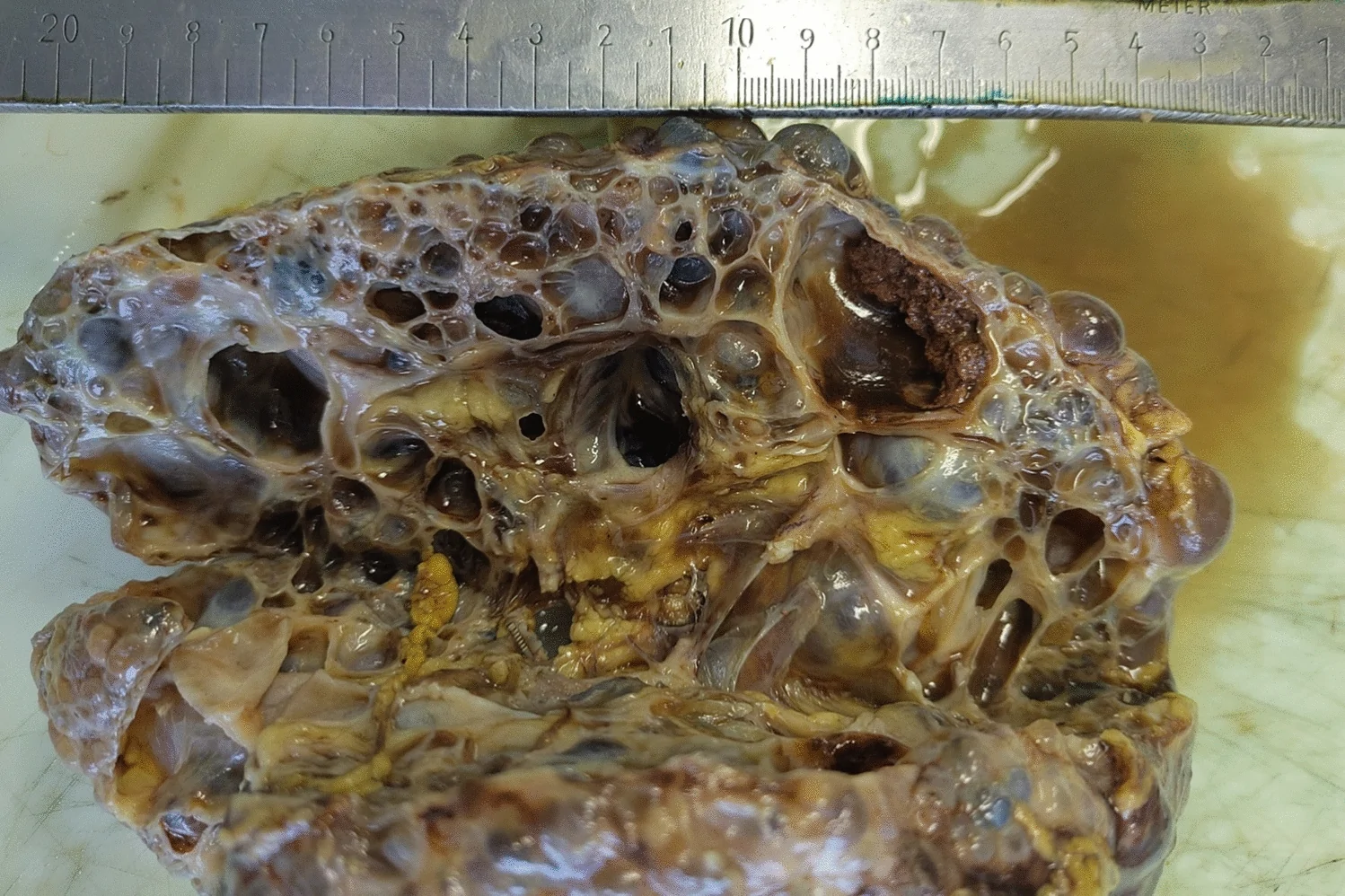
Nephrectomy specimen from a polycystic kidney. The renal parenchyma is replaced by multiple cysts of varying size, and the original architecture is no longer discernible. A larger cystic hemorrhage is located at the upper pole

Polycystic kidney disease is characterized by progressive cyst formation and distortion of the renal parenchyma, ultimately leading to chronic kidney disease and end-stage renal disease [1] (Fig. 1). Autosomal dominant polycystic kidney disease (ADPKD) is the most common inherited kidney disease, accounting for approximately 90% of cases. ADPKD affects approximately 1 in 400 to 1 in 1000 individuals worldwide and ranks as the fourth leading cause of end-stage renal disease (ESRD) in Europe. It accounts for approximately 10–15% of individuals requiring dialysis and 9–10% of those undergoing renal transplantation [2–4]. ADPKD symptoms typically manifest in adulthood, often between the ages of 30 and 40. Progressive renal disease occurs in 45% of patients by the age of 60 [1, 5]. Clinical manifestations, include pain, hematuria, recurrent infection, gastrointestinal compression symptoms, and several extra-renal manifestations [2, 5, 6]. While conservative medical therapy, including blood pressure control and symptomatic treatment, forms the cornerstone of ADPKD management, surgical interventions remain a common necessity for managing more severe complications. Around 20% of patients will eventually need to undergo nephrectomy in various clinical scenarios: uncontrolled pain or discomfort, recurrent infections or abscesses, and cases of life-threatening hemorrhage due to ruptured cysts [7–10].

The optimal timing of native nephrectomy relative to transplantation and the optimal operative approach remain debated. Open nephrectomy offers wide exposure, but is associated with substantial surgical trauma, whereas laparoscopic and robotic techniques may reduce postoperative pain and the length of hospital stay. [11–16] However, nephrectomy is a major surgery with potential risks, making careful patient selection and thorough preoperative evaluation essential for optimal outcomes. The present study aims to assess ADPKD patients’ pre-, peri-, and post-operative data following nephrectomy, analyze perioperative complications, and identify risk factors to improve surgical outcomes.

## Materials and methods

We retrospectively reviewed data from 135 episodes of native nephrectomy in patients with ADPKD at Hannover Medical School between October 2012 and October 2022. Eleven surgeries with incomplete records were excluded, leaving 124 nephrectomy episodes for analysis.

The indication was primarily symptom-driven and included recurrent cyst infection, pain or mass-related discomfort, recurrent hemorrhage or hematuria, recurrent urinary tract infection, and suspicion of malignancy. Size and space considerations were also taken into account, particularly when transplantation planning required adequate graft space. When more than one indication was present, the main indication recorded in the dataset reflected the dominant clinical reason prompting surgery. Open nephrectomy was the standard approach for larger or technically complex kidneys, whereas laparoscopic or robotic procedures were increasingly used for selected smaller kidneys as institutional experience evolved over time.

The timing of nephrectomy relative to transplantation was individualized. Pre-transplant nephrectomy was reserved for patients with severe or recurrent symptoms, uncontrolled infection or bleeding, suspicion of malignancy, or clinically relevant space restriction that could compromise graft implantation. In the absence of such factors, nephrectomy was generally deferred to avoid an additional major procedure before transplantation.

We collected demographic variables, comorbidities, operative characteristics, transplant and dialysis status, perioperative laboratory values, pathology, length of stay, transfusions, revision surgery, readmission, and perioperative complications graded according to Clavien–Dindo. Continuous variables are presented as mean ± standard deviation or median, and categorical variables as number (percentage). Logistic regression was used to assess predictors of overall and major complications. For the approach sensitivity analysis, operative specimen weight was defined as the removed kidney weight in unilateral or consecutive episodes and as the sum of both kidney weights in simultaneous bilateral nephrectomies. Operative specimen length was used as a secondary sensitivity descriptor, defined as the removed kidney length in unilateral or consecutive episodes and the maximum of both kidney lengths in simultaneous bilateral cases. Statistical analyses were performed using IBM SPSS Statistics v26.0. A two-sided *p* value < 0.05 was considered statistically significant.

## Results

The mean age was 56.06 years, and 37.9% of episodes occurred in female patients. Kidney transplantation was present in 40.3% of the cohort, 42.7% had polycystic liver disease, and 60.5% were on dialysis preoperatively. The most common main indications for nephrectomy were recurrent cystic infection (26.6%), pain or discomfort (16.9%), recurrent cystic hemorrhage (14.5%), recurrent urinary tract infection (10.5%), and suspected malignancy (10.5%).

Perioperative complications occurred in 73/124 episodes (58.9%), including 42 minor complications (33.9%) and 31 major complications (25.0%) (Table 1). The most frequent postoperative events were transfusion-requiring anemia, ileus, infection, hemorrhage, abscess formation, and sepsis. Intraoperative complications occurred in 16.1% of episodes and included bleeding requiring transfusion as well as gastrointestinal or vascular injury. Median hospital stay was 13 days (range 6–253), 19.4% of patients received postoperative transfusions, and 8.9% required revision surgery.

**Table 1:** Clavien–Dindo classification of perioperative complications

Variable	Value
Total	124
Perioperative complications	73 (58.9%)
Minor complications (Clavien-Dindo I–II)	42 (33.9%)
Grade I	10 (8.1%)
Grade II	32 (25.8%)
Major complications (Clavien-Dindo III–V)	31 (25.0%)
Grade III	25 (20.2%)
Grade IV	4 (3.2%)
Grade V	2 (1.6%)

No indication for surgery was significantly associated with overall complications (Table 2).

**Table 2:** Indications for nephrectomy and association with overall complications

Indication for nephrectomy	*n* (%)	*p* value
Recurrent cystic infection	33 (26.6%)	0.543
Pain or discomfort	21 (16.9%)	0.628
Recurrent cystic hemorrhage	18 (14.5%)	0.606
Recurrent UTI	13 (10.5%)	0.770
Suspected malignancy	13 (10.5%)	1.000
GI symptoms/early satiety	9 (7.3%)	1.000
Infection prophylaxis	5 (4.0%)	1.000
Dyspnea	5 (4.0%)	0.648
Recurrent hematuria	3 (2.4%)	0.067
Space for transplantation	2 (1.6%)	0.167
Others	2 (1.6%)	0.512

Most episodes were treated by an open approach (92.7%); 7 episodes were laparoscopic and 2 were robotic-assisted (Table 3). Open procedures were performed for substantially larger kidneys than minimally invasive procedures, with a median operative specimen weight of 3518.5 g versus 495.5 g (*p* < 0.001) and a median operative specimen length of 26.0 cm versus 15.5 cm (*p* < 0.001). Overall complication rates were 60.0% for open procedures and 44.4% for minimally invasive procedures (*p* = 0.486), whereas major complication rates were 27.0% and 0%, respectively (*p* = 0.110). However, after adjustment for operative specimen weight and bilateral status, minimally invasive surgery was not independently associated with a lower risk of overall complications (OR 0.75, 95% CI 0.15–3.79; *p* = 0.726).

**Table 3:** Surgical characteristics and perioperative course

Variable	Value
Total, *n*	124
Open nephrectomy	115 (92.7%)
Laparoscopic nephrectomy	7 (5.6%)
Robotic-assisted nephrectomy	2 (1.6%)
Unilateral nephrectomy	33 (26.6%)
Simultaneous bilateral nephrectomy	66 (53.2%)
Consecutive bilateral nephrectomy episodes	25 (20.2%)
Post-transplant nephrectomy	50 (40.3%)
Transperitoneal approach	120 (96.8%)
Intraoperative transfusion	18 (14.5%)
Postoperative ICU admission	79 (63.7%)
Postoperative transfusion	24 (19.4%)
Hospital readmission	7 (5.6%)
Revision surgery	11 (8.9%)

In the multivariable model for overall complications, lower preoperative hemoglobin independently increased risk (OR 0.74 per g/dL, 95% CI 0.61–0.89; *p* = 0.002), whereas left-sided current nephrectomy was associated with a lower overall complication rate (OR 0.25, 95% CI 0.09–0.72; *p* = 0.011). In the multivariable model for major complications, diverticulosis remained an independent risk factor (OR 5.03, 95% CI 1.76–14.33; *p* = 0.003), and higher intraoperative transfusion requirement was also associated with major complications (OR 1.52 per unit, 95% CI 1.06–2.18; *p* = 0.022). Greater operative specimen weight showed a trend toward higher major complication risk (OR 1.12 per kg, 95% CI 0.98–1.28; *p* = 0.085) (Table 4).

**Table 4:** Multivariable logistic regression models for complication outcomes

	Univariate		Multivariable (overall complications)			Multivariable (major complications)		
Variable	*p* value (overall complications)	*p* value (major complications)	OR	95% CI	*p* value	OR	95% CI	*p* value
Age	0.317	0.302	–	–	–	–	–	–
Sex	0.530	0.915	–	–	–	–	–	–
BMI	0.948	0.366	–	–	–	–	–	–
Kidney transplantation	0.593	0.143	–	–	–	–	–	–
Preoperative dialysis	0.752	0.342	–	–	–	–	–	–
Therapeutic anticoagulation	0.476	0.744	–	–	–	–	–	–
Previous abdominal surgery	0.901	0.338	–	–	–	–	–	–
Polycystic liver disease	0.056	0.753	–	–	–	–		–
Diverticulosis	0.129	0.016	–	–	–	5.47	1.78–16.80	**0.003**
Cardiovascular disease	0.836	0.613	–	–	–	–	–	–
Diabetes mellitus	0.492	0.822	–	–	–	–	–	–
Preoperative Hb, g/dL	0.007	0.062	0.72	0.60–0.88	**0.001**	0.79	0.62–1.01	0.056
Open nephrectomy	0.367	0.110*	–	–	–	–	–	–
Left-sided current nephrectomy	< 0.001	0.194	0.31	0.09–0.99	**0.047**	1.29	0.21–7.94	0.783
Simultaneous bilateral nephrectomy (vs consecutive bilateral vs unilateral; overall 3-group comparison)	**0.015**	0.258	–	–	–	–	–	–
Intraoperative transfusions, U	0.998	0.028	–	–	–	1.58	1.08–2.30	**0.018**
Operative specimen weight, kg	0.064	0.050	–	–	–	1.11	0.95–1.30	0.179
Malignancy	0.663	0.744	–	–	–	–	–	–

Bold value indicates statistical significance (*p* < 0.05)

When nephrectomy episodes were separated by laterality, unilateral nephrectomy accounted for 33 episodes (26.6%), simultaneous bilateral nephrectomy for 66 (53.2%), and consecutive bilateral nephrectomy episodes for 25 (20.2%). Complications occurred in 39.4% of unilateral episodes, 69.7% of simultaneous bilateral episodes, and 56.0% of consecutive bilateral episodes (*p* = 0.015). Major complications occurred in 15.2%, 30.3%, and 24.0% of these groups, respectively (*p* = 0.258).

Nephrectomies were more commonly performed post-transplant (40.3%) than pre-transplant (5.6%). Among the 57 episodes performed before or after transplantation, overall complication rates did not differ between pre-transplant and post-transplant nephrectomies (57.1% vs 56.0%; *p* = 1.000). Mean operative time was longer in pre-transplant nephrectomy (178.1 vs 124.5 min; *p* = 0.005), whereas length of stay and transfusion requirement did not differ significantly. Malignant findings were present in 11.2% of pathology specimens, most commonly clear-cell renal cell carcinoma.

## Discussion

### Perioperative morbidity, risk factors and complications

Recent studies on nephrectomy in patients with polycystic kidney disease have reported widely varying rates of perioperative complications, with most studies focusing primarily on the timing of nephrectomy in relation to renal transplantation. Reported complication rates range from 26.6 to 73%. [11, 17] In the present study, perioperative complications occurred in 58.9% of nephrectomy episodes and major complications in 25.0%. This relatively high morbidity underlines that native nephrectomy in ADPKD should remain restricted to carefully selected patients with clear clinical indications.

The role of comorbidity in perioperative risk remains insufficiently characterized in ADPKD-specific series. In our multivariable analysis, lower preoperative hemoglobin independently predicted overall complications, whereas diverticulosis was independently associated with major complications. Although causality cannot be inferred from this retrospective analysis, this finding is biologically plausible in a cohort treated almost exclusively through a transperitoneal approach, as diverticulosis may reflect more challenging colonic mobilization or greater susceptibility to bowel-related inflammatory or infectious complications. The association between operative complexity and outcome is further supported by the finding that greater operative specimen weight showed a trend toward higher major complication risk. Kidney size has long been regarded as a surrogate of disease burden in ADPKD and has also been linked to deterioration of renal function [18]. These findings suggest that presumed operative complexity, rather than kidney size alone, should be considered when assessing perioperative risk.

### Renal transplantation

The higher rate of post-transplant nephrectomy (40.3%) compared with pre-transplant nephrectomy (5.6%) in our cohort likely reflects the fact that many patients can proceed to transplantation without native nephrectomy and require later intervention only if symptoms or other kidney-related problems arise. At our institution, the timing of nephrectomy relative to transplantation was individualized. Pre-transplant nephrectomy was reserved for patients with compelling indications, such as severe or recurrent symptoms or clinically significant space restriction that could compromise graft implantation. In the absence of these factors, nephrectomy was generally deferred to preserve residual renal function and avoid an additional major procedure before transplantation.

In our series, complication rates were nearly identical in pre- and post-transplant nephrectomy episodes (57.1% vs. 56.0%, *p* = 1.000). Operative time was significantly longer in the pre-transplant group (178.1 vs. 124.5 min, *p* = 0.005), whereas length of stay and transfusion requirements did not differ significantly. These findings suggest that transplant chronology alone should not determine the indication for surgery. Instead, the decision should be guided primarily by symptoms and clinical necessity. This interpretation is partly consistent with prior reports showing no clear disadvantage of one strategy over the other, although other studies have associated pre-transplant nephrectomy with higher morbidity [17, 19]. In the series of Kirkman et al., complications were more frequent among pre-transplant nephrectomies [20]. Similarly, Chebib et al. reported a markedly lower complication rate in post-transplant nephrectomy than in pre-transplant nephrectomy (26.6% vs. 48%, *p* = 0.03) [11]. Based on previous studies, only a minority of ADPKD patients ultimately require nephrectomy, because many relevant complications arise after transplantation rather than before it [4, 11]. For this reason, several authors have advocated avoiding routine pre-transplant or simultaneous procedures unless clear indications are present [17, 20–22]. Our study did not include simultaneous nephrectomy with kidney transplantation. Nevertheless, Jänigen et al. showed in a matched-pair analysis of 193 ADPKD recipients that simultaneous ipsilateral nephrectomy during transplantation can be performed safely in selected patients and may reduce the total number of operations [23].

### Surgical approach

Choosing the optimal operative approach in ADPKD remains challenging. Open nephrectomy offers wide exposure and reliable vascular control, whereas laparoscopic and robotic techniques may reduce postoperative pain and accelerate recovery in selected patients. In our study, open surgery remained the predominant approach (92.7%), which reflects our institutional practice of reserving minimally invasive techniques for smaller and technically less complex kidneys. This selection bias was confirmed by the marked size difference between the groups: open procedures were performed for substantially larger kidneys than those in minimally invasive procedures.

Previous studies, including the meta-analysis by Guo et al., have suggested that laparoscopic nephrectomy can be a safe and effective alternative to open surgery in ADPKD, with lower morbidity and reduced blood loss in experienced hands [12]. In our series, 9 nephrectomy episodes were performed using minimally invasive techniques, including 7 laparoscopic and 2 robotic procedures. Descriptively, these cases showed lower crude rates of major complications and shorter hospital stays. However, this finding must be interpreted with caution because the minimally invasive subgroup was very small and highly selected. After adjustment for operative specimen weight and bilateral status, a minimally invasive approach was not independently associated with lower overall morbidity. Our data, therefore, do not support a causal claim that minimally invasive surgery reduces complications in ADPKD. Rather, they indicate that laparoscopic and robotic nephrectomy are feasible in carefully selected patients with smaller kidneys. This interpretation remains in line with ADPKD-specific reports suggesting comparable safety and shorter recovery in selected minimally invasive cases [11, 13–15]. Chebib et al. likewise emphasized that surgical modality should be selected according to individual anatomical and clinical factors [11]. Similar to the observations of Guo et al., their study suggested that laparoscopic nephrectomy can be performed safely even in enlarged polycystic kidneys, with the additional advantage of shorter hospitalization [11, 12]. In contrast, Lubennikov et al., who reported comparable outcomes between open and laparoscopic bilateral nephrectomy, suggested that open surgery may remain preferable in patients with massively enlarged kidneys that require wide access [16]. More recent reviews of robotic surgery provide broader contextual support for the increasing use of robotic platforms, improved visualization, and greater technical dexterity, although not specific evidence in ADPKD [24].

To contextualize our findings, Table 5 summarizes selected studies cited in the present manuscript that report complication-related outcomes after open, laparoscopic, hand-assisted laparoscopic, and robotic-assisted nephrectomy.

**Table 5:** Selected studies reporting complication-related outcomes after nephrectomy in ADPKD

Study	Design/cohort	Approach compared	Sample size	Complication-related findings
Guo et al. [12]	Systematic review and meta-analysis in ADPKD	Laparoscopic nephrectomy (LN) vs open nephrectomy (ON)	7 studies; 195 cases (118 LN/77 ON)	LN was associated with lower overall complications (RR 0.545, 95% CI 0.329–0.903; *p* = 0.018), lower transfusion requirement (RR 0.345, 95% CI 0.183–0.650; *p* = 0.001), less blood loss (WMD − 180.245 mL; *p* = 0.010), and shorter length of stay (WMD − 3.576 days; *p* < 0.001), but longer operative time (WMD 30.236 min; *p* < 0.001)
Chebib et al. [11]	Retrospective transplant-recipient cohort with ADPKD	Pre-transplant vs post-transplant nephrectomy; open and laparoscopic techniques both represented	114 nephrectomies among 470 transplant recipients	Complications were less common post-transplant than pre-transplant (26.6% vs 48.0%; *p* = 0.03). Nephrectomy was done laparoscopically in 31% of pre-transplant vs 86% of post-transplant cases. Complication rates were reported as similar by surgical technique (open 33.3% vs laparoscopic 33.0%; *p* = 0.66)
Eng et al. [15]	Retrospective comparative series in ADPKD	Hand-assisted laparoscopic nephrectomy (HALN) vs open nephrectomy	78 patients identified; 56 HALN, 18 open, 2 conversions noted in accessible snippet	HALN was reported to result in shorter hospital stay and reduced need for transfusion than open nephrectomy
Lubennikov et al. [16]	Retrospective single-center bilateral nephrectomy series in ADPKD with ESRD	Laparoscopic bilateral nephrectomy vs open midline laparotomy	108 patients(36 laparoscopic/72 open)	Postoperative complications were reported as equal with both approaches; postoperative mortality occurred only in the emergent group. Laparoscopic surgery had longer operative time but faster rehabilitation and shorter hospital stay

### Surgical side and bilateral strategy

Although right-sided unilateral nephrectomy is sometimes considered technically more straightforward because of vascular anatomy, the operative side in ADPKD is ultimately determined by symptom distribution and anatomical considerations [20]. In our series, left-sided current nephrectomy was associated with a lower risk of overall complications in multivariable analysis. One possible explanation is the technical difficulty of right-sided nephrectomy in the presence of marked hepatomegaly or polycystic liver disease, which is common in ADPKD and may complicate exposure and dissection [6].

After classification into unilateral, simultaneous bilateral, and consecutive bilateral nephrectomy episodes, simultaneous bilateral nephrectomy showed the highest overall complication burden, while consecutive bilateral episodes had intermediate complication rates. Complications occurred in 39.4% of unilateral episodes, 69.7% of simultaneous bilateral episodes, and 56.0% of consecutive bilateral episodes (*p* = 0.015). Major complications occurred in 15.2%, 30.3%, and 24.0% of these groups, respectively (*p* = 0.258). These findings are consistent with the study by Fuller et al., which reported that simultaneous bilateral nephrectomy was associated with increased transfusion requirements and less favorable post-transplant outcomes compared with staged procedures [7]. Some authors have advocated a bilateral strategy to prevent future post-transplant complications arising from retained native kidneys, especially under immunosuppression [21, 22, 25].

### Renal functional considerations

Renal functional consequences also merit consideration when counseling patients. Although functional outcomes were not primary endpoints of the present study, recent multicenter data outside the ADPKD setting show that postoperative acute kidney injury and new-onset clinically significant chronic kidney disease are not negligible after nephrectomy, particularly when operative complexity is higher or radical nephrectomy is performed [26]. Similarly, the RENSAFE model identified preoperative renal function, diabetes, and surgical modality as major determinants of postoperative acute kidney injury and chronic kidney disease stage ≥ 3b after nephrectomy [27]. These studies are not directly transferable to ADPKD, but they support a general principle that nephron loss and operative complexity can have meaningful functional consequences and reinforce the need for strict indications in patients who still have residual renal function.

### Malignancy

Histopathological examination revealed malignant findings in 11.2% of nephrectomy specimens, which is higher than expected in the general pre-transplant ESRD population [28]. Of these, 10 patients (8.0%) underwent surgery because malignancy was suspected on preoperative imaging, whereas in 4 patients (3.2%) malignancy was detected incidentally. Our findings are in keeping with the report by Hajj et al., who observed a malignancy prevalence of 12.4% in ADPKD patients with chronic renal failure [29]. Other studies have also reported elevated rates of renal neoplasia in ADPKD surgical specimens, including 5.2% in the series by Kirkman et al. and 5.3% in the study by Jilg et al [20, 30].

### Limitations

Our study has several limitations. It is retrospective and single-center. The minimally invasive subgroup was very small, and confounding by indication was substantial because smaller kidneys were preferentially selected for minimally invasive surgery. Not all stages of consecutive bilateral nephrectomy were available within the study interval. In addition, long-term functional outcomes and transplantation-related late events were not systematically captured. These limitations constrain causal inference and generalizability.

## Conclusion

Definitive guidelines for managing complicated cases of ADPKD are not well-established, leaving the decision-making mainly reliant on the expertise of surgeons. The results of our study demonstrate that nephrectomy in ADPKD is a procedure with a relatively high perioperative morbidity rate (58.9%). However, most of these were short-term complications and were satisfactorily managed postoperatively resulting in an acceptable rate of readmission and mortality. We emphasize that native nephrectomy should be performed exclusively with strict indications and not routinely due to the surgical complications and risks. Thus, the timing and indication to perform nephrectomy should primarily be determined by the severity of symptoms rather than their sequence in relation to transplantation. Minimally invasive nephrectomy may be feasible in selected patients with smaller kidneys; however, our limited data do not demonstrate an independent reduction in morbidity after adjustment for case complexity. This question should be addressed in larger cohorts of patients undergoing minimally invasive nephrectomy.

## Data Availability

The clinical data supporting the findings of this study are available from Hannover Medical School but restrictions apply due to ethical and privacy considerations. Data are available from the corresponding author upon reasonable request and with permission from the institutional review board.

## References

[1] Chebib FT, Torres VE (2016) Autosomal dominant polycystic kidney disease: core curriculum 2016. Am J Kidney Dis 67(5):792–810. 10.1053/j.ajkd.2015.07.037 26530876 PMC4837006

[2] Harris PC, Torres VE (1993) Polycystic kidney disease, autosomal dominant. GeneReviews. Seattle (WA): University of Washington, Seattle 20301424

[3] Spithoven EM, Kramer A, Meijer E et al (2014) Analysis of data from the ERA-EDTA Registry indicates that conventional treatments for chronic kidney disease do not reduce the need for renal replacement therapy in autosomal dominant polycystic kidney disease. Kidney Int 86(6):1244–1252. 10.1038/ki.2014.120 24827775

[4] Argyrou C, Moris D, Vernadakis S (2017) Tailoring the “perfect fit” for renal transplant recipients with end-stage polycystic kidney disease: indications and timing of native nephrectomy. In Vivo 31(3):307–312. 10.21873/invivo.11060 28438856 PMC5461438

[5] Harris PC, Torres VE (2009) Polycystic kidney disease. Annu Rev Med 60:321–337. 10.1146/annurev.med.60.101707.125712 18947299 PMC2834200

[6] Luciano RL, Dahl NK (2014) Extra-renal manifestations of autosomal dominant polycystic kidney disease (ADPKD): considerations for routine screening and management. Nephrol Dial Transplant 29(2):247–254. 10.1093/ndt/gft437 24215018

[7] Fuller TF, Brennan TV, Feng S et al (2005) End stage polycystic kidney disease: indications and timing of native nephrectomy relative to kidney transplantation. J Urol 174(6):2284–2288. 10.1097/01.ju.0000181208.06507.aa 16280813

[8] Neeff HP, Pisarski P, Tittelbach-Helmrich D et al (2013) One hundred consecutive kidney transplantations with simultaneous ipsilateral nephrectomy in patients with autosomal dominant polycystic kidney disease. Nephrol Dial Transplant 28(2):466–471. 10.1093/ndt/gfs118 23042709

[9] Jankowska M, Kuźmiuk-Glembin I, Skonieczny P, Dębska-Ślizień A (2018) Native nephrectomy in renal transplant recipients with autosomal dominant polycystic kidney disease. Transplant Proc 50(6):1863–1867. 10.1016/j.transproceed.2018.02.100 30056917

[10] Jean RA, Alexandre M, Yoo PS (2018) Kidney transplantation with and without native nephrectomy for polycystic kidney disease: results of the National Inpatient Sample and the rationale for a 2-staged procedure. J Am Coll Surg 226(6):1079–1084. 10.1016/j.jamcollsurg.2017.11.021 29224797

[11] Chebib FT, Prieto M, Jung Y et al (2015) Native nephrectomy in renal transplant recipients with autosomal-dominant polycystic kidney disease. Transplant Direct 1(10):e43. 10.1097/TXD.0000000000000554 26981586 PMC4788702

[12] Guo P, Xu W, Li H, Ren T, Ni S, Ren M (2015) Laparoscopic nephrectomy versus open nephrectomy for patients with autosomal dominant polycystic kidney disease: a systematic review and meta-analysis. PLoS ONE 10(6):e0129317. 10.1371/journal.pone.0129317 26053633 PMC4460089

[13] Lucas SM, Mofunanya TC, Goggins WC, Sundaram CP (2010) Staged nephrectomy versus bilateral laparoscopic nephrectomy in patients with autosomal dominant polycystic kidney disease. J Urol 184(5):2054–2059. 10.1016/j.juro.2010.06.150 20850813

[14] Whitten MG, Van der Werf W, Belnap L (2006) A novel approach to bilateral hand-assisted laparoscopic nephrectomy for autosomal dominant polycystic kidney disease. Surg Endosc 20(4):679–684. 10.1007/s00464-005-0229-z 16432653

[15] Eng M, Jones CM, Cannon RM, Marvin MR (2013) Hand-assisted laparoscopic nephrectomy for polycystic kidney disease. JSLS 17(2):279–284. 10.4293/108680813X13654754535719 23925022 PMC3771795

[16] Lubennikov AE, Petrovskii NV, Krupinov GE et al (2021) Bilateral nephrectomy in patients with autosomal dominant polycystic kidney disease and end-stage chronic renal failure. Nephron 145(2):164–170. 10.1159/000513168 33550285 PMC8006584

[17] Patel P, Horsfield C, Compton F, Taylor J, Koffman G, Olsburgh J (2011) Native nephrectomy in transplant patients with autosomal dominant polycystic kidney disease. Ann R Coll Surg Engl 93(5):391–395. 10.1308/003588411X582690 21943464 PMC3365458

[18] Grantham JJ, Torres VE, Chapman AB et al (2006) Volume progression in polycystic kidney disease. N Engl J Med 354(20):2122–2130. 10.1056/NEJMoa054341 16707749

[19] Akoh JA (2015) Current management of autosomal dominant polycystic kidney disease. World J Nephrol 4(4):468–479. 10.5527/wjn.v4.i4.468 26380198 PMC4561844

[20] Kirkman MA, van Dellen D, Mehra S et al (2011) Native nephrectomy for autosomal dominant polycystic kidney disease: before or after kidney transplantation? BJU Int 108(4):590–594. 10.1111/j.1464-410X.2010.09938.x 21166760

[21] Rozanski J, Kozlowska I, Myslak M et al (2005) Pretransplant nephrectomy in patients with autosomal dominant polycystic kidney disease. Transplant Proc 37(2):666–668. 10.1016/j.transproceed.2004.12.115 15848495

[22] Brazda E, Ofner D, Riedmann B, Spechtenhauser B, Margreiter R (1996) The effect of nephrectomy on the outcome of renal transplantation in patients with polycystic kidney disease. Ann Transplant 1(2):15–18 (**DOI not verified**) 9869924

[23] Jänigen BM, Hempel J, Holzner P et al (2020) Simultaneous ipsilateral nephrectomy during kidney transplantation in autosomal dominant polycystic kidney disease: a matched pair analysis of 193 consecutive cases. Langenbecks Arch Surg 405(6):833–842. 10.1007/s00423-020-01939-3 32705344 PMC7471159

[24] Bignante G, Orsini A, Lasorsa F et al (2024) Robotic-assisted surgery for the treatment of urologic cancers: recent advances. Expert Rev Med Devices 21(12):1165–1177. 10.1080/17434440.2024.2435546 39618104

[25] Skauby MH, Øyen O, Hartman A, Leivestad T, Wadström J (2012) Kidney transplantation with and without simultaneous bilateral native nephrectomy in patients with polycystic kidney disease: a comparative retrospective study. Transplantation 94(4):383–388. 10.1097/TP.0b013e31825812b9 22828736

[26] Pecoraro A, Roussel E, Amparore D et al (2023) New-onset chronic kidney disease after surgery for localised renal masses in patients with two kidneys and preserved renal function: a contemporary multicentre study. Eur Urol Open Sci 52:100–108. 10.1016/j.euros.2023.04.011 37284048 PMC10240519

[27] Saitta C, Afari JA, Autorino R et al (2023) Development of a novel score (RENSAFE) to determine probability of acute kidney injury and renal functional decline post surgery: a multicenter analysis. Urol Oncol 41(12):487.e15-487.e23. 10.1016/j.urolonc.2023.09.015 37880003

[28] Breda A, Luccarelli G, Rodriguez-Faba O et al (2015) Clinical and pathological outcomes of renal cell carcinoma (RCC) in native kidneys of patients with end-stage renal disease: a long-term comparative retrospective study with RCC diagnosed in the general population. World J Urol 33(1):1–7. 10.1007/s00345-014-1248-y 24504760

[29] Hajj P, Ferlicot S, Massoud W et al (2009) Prevalence of renal cell carcinoma in patients with autosomal dominant polycystic kidney disease and chronic renal failure. Urology 74(3):631–634. 10.1016/j.urology.2009.02.078 19616833

[30] Jilg CA, Drendel V, Bacher J et al (2013) Autosomal dominant polycystic kidney disease: prevalence of renal neoplasias in surgical kidney specimens. Nephron Clin Pract 123(1–2):13–21. 10.1159/000351049 23752029

